# Risk factors associated with 31-day unplanned hospital readmission in newborns: a systematic review

**DOI:** 10.1007/s00431-023-04819-2

**Published:** 2023-01-27

**Authors:** Phillip R. Della, Haichao Huang, Pamela A. Roberts, Paul Porter, Elizabeth Adams, Huaqiong Zhou

**Affiliations:** 1 Curtin School of Nursing, Curtin University, GPO Box U 1987, Perth, Western Australia 6845 Australia; 2 School of Nursing, Tianjin University of Traditional Chinese Medicine, Tianjin, China; 3 Joondalup Health Campus, Joondalup, Western Australia Australia; 4 European Federation of Nurses Associations, Clos du Parnasse, Brussels, 11A B-1050 Belgium; 5 General Surgical Ward, Perth Children’s Hospital, Nedlands, Western Australia Australia

**Keywords:** Newborn, 31-day unplanned hospital readmission, Risk factors

## Abstract

The purpose of this study is to synthesize evidence on risk factors associated with newborn 31-day unplanned hospital readmissions (UHRs). A systematic review was conducted searching CINAHL, EMBASE (Ovid), and MEDLINE from January 1st 2000 to 30th June 2021. Studies examining unplanned readmissions of newborns within 31 days of discharge following the initial hospitalization at the time of their birth were included. Characteristics of the included studies examined variables and statistically significant risk factors were extracted from the inclusion studies. Extracted risk factors could not be pooled statistically due to the heterogeneity of the included studies. Data were synthesized using content analysis and presented in narrative and tabular form. Twenty-eight studies met the eligibility criteria, and 17 significant risk factors were extracted from the included studies. The most frequently cited risk factors associated with newborn readmissions were gestational age, postnatal length of stay, neonatal comorbidity, and feeding methods. The most frequently cited maternal-related risk factors which contributed to newborn readmissions were parity, race/ethnicity, and complications in pregnancy and/or perinatal period.

*Conclusion*: This systematic review identified a complex and diverse range of risk factors associated with 31-day UHR in newborn. Six of the 17 extracted risk factors were consistently cited by studies. Four factors were maternal (primiparous, mother being Asian, vaginal delivery, maternal complications), and two factors were neonatal (male infant and neonatal comorbidities). Implementation of evidence-based clinical practice guidelines for inpatient care and individualized hospital-to-home transition plans, including transition checklists and discharge readiness assessments, are recommended to reduce newborn UHRs.

**Table:** 

**What is Known:** *• Attempts have been made to identify risk factors associated with newborn UHRs; however, the results are inconsistent.*	
**What is New:** *• Six consistently cited risk factors related to newborn 31-day UHRs. Four maternal factors (primiparous, mother being Asian, vaginal delivery, maternal complications) and 2 neonatal factors (male infant and neonatal comorbidities).* *• The importance of discharge readiness assessment, including newborn clinical fitness for discharge and parental readiness for discharge. Future research is warranted to establish standardised maternal and newborn-related variables which healthcare providers can utilize to identify newborns at greater risk of UHRs and enable comparison of research findings.*

**Supplementary Information:**

The online version contains supplementary material available at 10.1007/s00431-023-04819-2.

Newborn unplanned hospital readmission (UHR) is defined as an unexpected hospital readmission within a specified time period following discharge from the initial hospitalization at the time of birth [1, 2]. Newborn UHRs are widely recognized as indicators of health service delivery quality and contribute to neonatal morbidity and increased healthcare cost [3–5]. Some unplanned hospital readmissions may present due to incomplete or inappropriate transitional care at the time of discharge [6]. Others are related to risk factors such as feeding issues or prolonged jaundice that may have been preventable with an individualized hospital-to-home transition plan and improved transitional care [7, 8].

Identifying risk factors associated with UHRs of the newborn can assist in reducing readmission rates through improvements in clinical practice, policy development, and the use of maternal-child healthcare services. While studies have examined causes associated with neonatal morbidity and mortality [9, 10], there is no published review of risk factors associated with UHRs for the newborn. This paper systematically reviewed current literature identifying risk factors associated with newborn 31-day UHRs. The objectives were to review the characteristics of included studies and synthesize the identified risk factors related to newborn UHRs.

## Methods

The systematic review followed the 2009 PRISMA (Preferred Reporting Items for Systematic Reviews and Meta-Analysis) Statement [11].

### Data sources and search strategy

An electronic database search was carried out using the CINAHL, EMBASE (Ovid), MEDLINE from 1st January 2000 to 30th June 2021 with key search terms (“Readmission” or rehospitali* or readmission* or readmit* or re-admission*) and (newborn* or new born* or newly born or baby* or babies or premature or prematurity or preterm or pre term or preemie* or premie* or low birth weight or low birthweight (LBW) or very low birthweight (VLBW) or extremely low birth weight (ELBW or infant* or infancy or neonat*) (A complete search strategy is provided in Appendix 1).

Four inclusion criteria for this review were (1) primary research studies, (2) UHRs assessment/measurements within 31 days, (3) study design stated clearly and reported statistical analysis procedure/s, and (4) published in peer-reviewed journals and the English language with full text available. Studies were excluded when mixed adverse outcomes, including complications and emergency department (ED) visits post-hospital discharge or readmission were measured more than once. Conference abstract-only references were also excluded.

## Study selection

Two reviewers initially read all titles and abstracts independently to assess potential inclusion. Included full-text articles were then assessed against the inclusion criteria. Disagreements between the reviewers on potential articles for inclusion were resolved through discussion. Reference lists of all included articles were screened to identify additional articles.

## Data extraction

Data extraction included study characteristics, examined variables, and statistically significant risk factors. Study characteristics included study setting, population, sample size, the timing of data collection, study design, data source, readmission rate, and statistical analysis test/s used to identify risk factors as per Table 1. All examined variables or confounding factors and statistically significant risk factors were extracted as per Table 2.

**Table 1:** Characteristics of the 28 included studies

**Reference**	**Medical condition**	**Outcome measures**	**Study design**	**Data source**	**Sample size**	**Age**	**Follow-up period**	**Proportion readmitted**	**Data analysis**
Hensman et al. [27] USA	Healthy term infants	28-Day UHRs	A nested case–control Retrospective	A women and Infants Hospital Electronic medical records	UHR = 130; Matched controls = 260	37^0/7^–41^6/7^ GA	Jan 2016 to May 2017	2.2%	Hierarchical conditional logistic regression
Lee et al. [23] Canada	Healthy term singleton infants	7-Day UHRs	Retrospective	Administrative health databases— Province of Alberta	Model development: Vaginal delivery = 3378; Caesarean = 21,225; Validation: vaginal delivery = 21,583; Caesarean = 7477	Term newborns	2012 to 2015	3.3% by Vaginal delivery; 2.1% by caesarean	Multivariable backward stepwise logistic regression
Cools et al. [20] USA	Myelomeningocele repair newborns	30-Day UHRs	Retrospective Matched case–control	A single academic medical centre Medical records	58 Newborns (24 prenatal repair + 34 postnatal repair)	Prenatally repaired vs. postnatally: 32^1/7^vs.36^6/7^ GA	2011 to 2017	5% (pre) 39% (post)	*t*-tests
Fein et al. [40] USA	Term newborns after intensive phototherapy	30-Day UHRs	Retrospective	New York’s State Inpatient Database	94,626 Newborns	37^0/7^–42^6/7^ GA	2005 to 2011	3.0–3.1%	Multivariable regression
Kosowan et al. [41] Canada	Newborns	28-Day UHRs	Retrospective	Manitoba Centre for Health Policy houses	71,836 Infants	Newborn	2000 to 2010	10.32%	Logistic regression
Mallick et al. [25] India	Late preterm babies	31-Day UHRs	Prospective	A tertiary hospital Clinical record and telephone review	238 (LP SGA = 72; LP AGA = 166)	34^0/7^– 36^6/7^ GA	Nov 2011 to June 2015	19.4% 8.4%	Chi-square test Fisher’s exact test
Reed et al. [24] France	Preterm babies	30-Day UHRs	Prospective	25 regions of France Interviews, questionnaires, survey, and clinical record	3841	22–34 GA	March 2011 to Dec 2011	9.1%	Logistic regression
Yu et al. [26] USA	Neonatal hyperbilirubinemia newborns	30-Day UHRs	Retrospective Matched case–control	HealthCare Integrated Research Database	1,373 newborns with haemolytic NHB Vs. 1373 Non-haemolytic NHB	Newborns ≥ 35 GA	Jan 2011 to Aug 2017	8.7%	Paired *t*-tests
Bentz et al. [28] USA	Hyperbilirubinemia	30-Day UHRs	Retrospective	An urban children’s hospital Electronic medical records	653	< 37 GA vs ≥ 37 GA	2014 to 2016	16.1%	Multivariable regression
Flaherman et al. [29] USA	Newborn	30-Day UHRs	Retrospective	14 hospitals Electronic medical records	143,889 (108,745 vaginal births vs. 35,144 caesarean births)	≥ 36 GA Median of 39 GA	2009 to 2013	1.5% to 4.3%	Logistic regression
Harron et al. [30] UK	Newborn	30-Day UHRs	Retrospective	Population-level hospital data on births	4,667,827 Infants	Newborn (< 37 GA)	2005 to 2014	5.2%	Poisson generalised linear models
Patrick et al. [31] USA	Newborn NAS	30-Day UHRs	Retrospective	State Inpatient Database	700,613 Uncomplicated 51,748 Late preterm 1,643 NAS	Newborn	2006 to 2009	3% UC; 3.7% LP; 1.9% NAS	Logistic regression
Lain et al. [32] Australia	Term newborn Jaundice	14-Day UHRs	Retrospective	State-wide hospitals’ admission data	781,074 infants	Term newborn	2001 to 2010	0.8%	Logistic regression
Macdonald et al. [18] Canada	Infants born to women with and without HIV	30-Day UHRs	Retrospective	6 State level healthcare databases	1,133,505 pregnancies (634 living with HIV)	Newborn	Apr 2002 to Mar 2011	With HIV: 2.6% without HIV 3.7%	Generalized estimating equations
Moyer et al. [33] USA	Late-preterm newborn	28-Day UHRs	Retrospective	8 Regional hospitals Administrative database + Medical records	1,861	34–36 GA	2009	3.6%	Multivariable regression
Goyal et al. [22] USA	Late-preterm newborn delivered vaginally	7-Day UHRs	Retrospective	State-wide Hospital discharge data with vital records	161,200 patients 122 matched pairs based on LOS	34–36 GA	1993 to 2005	3.3%	Logistic regression
Young et al. [34] USA	Newborn with LOS ≤ 24 h	28-Day UHRs	Retrospective	21 Maternity services Electronic health database	5,308 Newborns	34–42 GA	2000 to 2010	0.79% to 4.46%	Multivariate logistic regression
Farhat and Rajab [35] Lebanon	Newborn	14-Days UHRs	Prospective	A general hospital investigation by caregiver and clinical records	478 Patients	Newborn	Sept 2009 to March 2010	7.9%	Logistic regression
Tseng et al. [36] Taiwan	Preterm -LBW	15- or 31-Day UHRs	Retrospective	National Health Insurance claim data	18,421 Patients	< 28 GA or 28–36 GA	2000 to 2002	9.6% (15d) 13.5% (31d)	Cox proportional hazard model
Tomashek et al. [37] USA	late preterm newborns	28-Day UHRs	Retrospective	State-wide linked database	1004 (34–36 GA) 24,320 (37–41 GA)	34–41 GA	1998 to 2002	LPN (3.5%) Term infant (2.0%)	Forward modified Poisson regression
Shapiro-Mendoza et al. [3] USA	Singleton late preterm infants vaginally delivered	28-Day UHRs	Retrospective	State-wide linked database	9552 Late preterm ‘healthy’ infants	34–36^6/7^ GA	1998 to 2002	4.8%	Poisson regression
Paul et al. [7] USA	Newborn	1^st^ 10 days of life UHRs	Retrospective	Birth records database	407,826 Patients	≥ 35 GA	1998 to 2002	0.6%	Logistic regression
Mackie et al. [2] USA	Cardiac surgery	30-day UHRs	Retrospective	A children’s hospital Medical records	162 Patients	Neonates	1992 to 2002	29.6%	Multivariate regression
Escobar et al. [38] USA	Newborn > 2000gm Dehydration	15-Day UHRs	Retrospective	11 Hospitals Administrative database and Medical records	51,383 Patients	≥ 36 GA	1995 to 1996	0.21%	Multivariate analysis
Geiger et al. [19] USA	Normal vaginal birth Jaundice	14-Day UHRs	Retrospectivecase–control	10 KPSC medical centres Electronic medical database	68,793 Vaginal deliveries	Newborn	1992 to 1994	0.2%	Logistic regression
Sword et al. [21] USA	Newborn	28-Day UHRs	Prospective	Survey & Structured telephone interview – 5 sites and Clinical records	1250 Survey; 875 interviews	Newborn	Nov 1998 to Jan 1999	2.4–6.7%	Logistic regression
Danielsen et al. [39] USA	Newborn	28-Day UHRs	Retrospective	State-wide linked database of Birth certificate, Newborn and Maternal hospitalisation records	1,214,545 Newborns	Newborn	1992 to 1995	2.76–3.02%	Logistic regression
Oddie et al. [17] UK	Newborn	28-Day UHRs	Retrospective	Five large maternity units Administrative database	32,015 Newborns	≥ 35 GA	1998	2.8%	Logistic regression

*GA* gestational age, *SGA* small for gestational age, *AGA* appropriate for gestational age, *LP* late preterm, *LP SGA* late preterm and SGA newborn, *LP AGA* late preterm and AGA newborn, *NAS* neonatal abstinence syndrome

**Table 2:** Seventeen significant risk factors associated with newborn 31-day UHRs

Reference number		**26**	**28**	**29**	**30**	**31**	**32**	**33**	**22**	**18**	**34**	**35**	**36**	**37**	**3**	**7**	**2**	**38**	**19**	**21**	**39**	**20**	**40**	**41**	**25**	**24**	**17**	**27**	**23**
Examined variables (*n* =)		1	5	3	16	10	12	14	1	10	4	9	10	2	13	17	15	16	12	16	11	1	8	10	1	19	9	24	8
Significant risk factors (*n* =)		1	4	2	8	3	9	5	0	0	2	5	4	1	4	8	2	7	6	2	6	1	6	2	1	1	4	5	7
Maternal-related risk factor	Primiparous mother		✔		✔		✔								✔	✔		✔	✔		✔								✔
	Maternal complications				✔		✔					✔			✔	✔			✔	✔	✔							✔	
	Maternal race		✔		✔		✔									✔		✔	✔		✔		✔						
	Insurance/income					✔									✔					✔	✔		✔						
	Maternal age						✔									✔		✔						✔					✔
	Geography of birth hospital/residence												✔										✔	✔			✔		✔
Newborn-related risk factor	Gestational age		✔		✔	✔	✔				✔	✔	✔	✔		✔		✔	✔						✔	✔	✔	✔	✔
	Comorbidity	✔	✔		✔	✔		✔					✔				✔		✔			✔	✔					✔	
	Length of stay				✔		✔	✔			✔	✔				✔	✔	✔			✔								✔
	Feeding method			✔			✔	✔				✔			✔			✔	✔								✔		
	Infant gender						✔						✔			✔		✔			✔		✔						✔
	Birth weight			✔	✔			✔																			✔		
	Delivery mode						✔					✔				✔												✔	
	Level of care							✔															✔						
	Formula received																											✔	
	Season of birth				✔																								
	Apgar score																												✔

## Quality assessment

The methodological quality of included studies was assessed independently by two reviewers using a standardised set of predefined criteria in six dimensions (Study participation, Study attrition, Prognostic factor measurement, Outcome measurement, Confounding measurement and account, and Analysis). The evaluation results of each item were rated as Yes/Partly/No/Unsure. The potential bias of each study was evaluated by overall risk “low” or “high” [12, 13].

## Data synthesis

Pooling extracted risk factors is not possible due to the heterogeneity of included studies such as diagnosis, examined variables, or follow-up period to identify readmissions. Therefore, content analysis was used to synthesize the extracted risk factors, and the results are presented narratively [11]. Due to the complex and diverse nature of the population and risk factors associated with newborn 31-day UHRs, it was decided on the collation of all available evidence as it is not possible to proceed with meaningful sub-analysis given the limited amount of research evidence available in sub-groups. The overall aim of this review was the identification of commonly cited risk factors and to promote awareness for healthcare providers to be able to recognize newborns at greater risk for UHR.

## Results

**Fig. 1:**
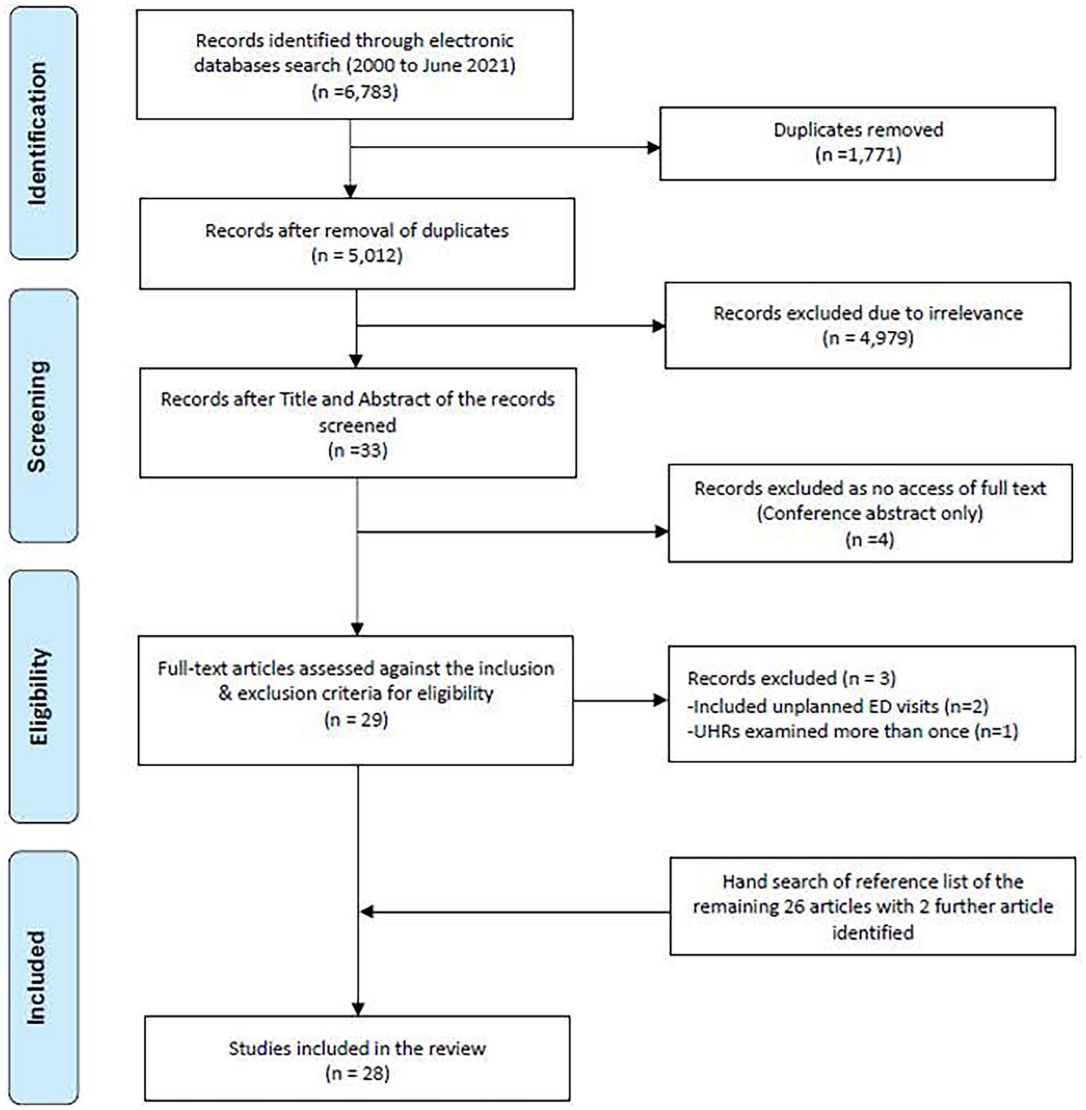
Flow chart for the search and study selection process (PRISMA)

A total of 6783 records were initially identified, after removing 1771 duplicates, 5012 records remained and were screened through titles and abstracts. Of these, 4979 records were excluded due to irrelevance and 33 relevant references were considered eligible for potential inclusion. A further 4 were excluded as they were conference abstracts only. A total of 29 references were retrieved as full text. Three studies were further excluded for the following reasons: (1) Outcome measures included unplanned ED visits [14, 15] (*n* = 2); (2) readmissions were measured more than once [16] (*n* = 1). Two additional articles [17, 18] were identified during a hand search of the reference lists. As a result, 28 studies were included in the systematic review. Figure 1 illustrates the search result and selection process.

## Study quality appraisal

Overall, the risk of potential bias for the 28 included studies was low against the six predefined dimensions of potential bias [12, 13]. Key characteristics of included populations were described clearly, samples were represented completely, all independent variables of the study population were measured appropriately, outcome variables of UHR were measured accurately, and statistical analysis tests were appropriate for the study design.

## Characteristics of the included studies

Characteristics of the 28 included studies are summarized in Table 1. Eighteen studies were conducted in the United States of America (USA), three from Canada, two from the United Kingdom (UK), and one from Australia, France, India, Lebanon, and Taiwan. Length of time between discharge from initial admission at the time of birth to unplanned readmission varied from 7 to 31 days. Twenty included studies used 28-day or 30-day UHRs. Seven of the 28 studies examined a combination of data from an administrative database and medical records; 13 used medical records only, while the remaining seven used administrative databases.

Twenty-two included studies retrieved data from multiple sites, while 6 from a single center. Samples sizes varied from 58 to 4,667,827 and UHRs rates varied from 0.2 [19] to 39% [20]. The majority of included studies (*n* = 18) recorded the age of patients using gestational age (GA), while nine studies referred to the newborn without specific GA. The four main types of the population involved in the 28 included studies were health-term newborns (*n* = 2), all live newborns (*n* = 9), late-preterm newborns with various health condition focus (*n* = 5), and newborn with varieties of health issues (*n* = 12).

The time span for the retrieved data varied from 2 months [21] to 12 years [22]. In particular, six included studies accessed over 10 years of data. Two of the 28 included studies reported risk predictive models of performance. All five of the identified predictive models in the studies demonstrated sub-optimal performance (C-statistic of 0.62, 0.69, and 0.62, respectively) [23, 24].

## Examined variables/confounding factors and significant risk factors

Variables or confounding factors differed across the 28 included studies. The number of examined variables for each study ranged from one [20, 22, 25, 26] to twenty-four [27]. Two studies [18, 22] reported inconclusive findings. Seventeen statistically significant risk factors related to newborn UHRs were extracted and grouped under either maternal or newborn-related factors in Table 2.

## Maternal-related risk factors

Seventeen of the 28 included studies identified maternal variables contributing to 31-day newborn UHRs. The three most frequently cited risk factors were maternal parity, pre-existing or perinatal complications, and race/ethnicity. Nine studies [3, 7, 19, 23, 28, 30, 32, 38, 39] identified the primiparous mother as a significant risk factor for readmission for newborns with an odds ratio (OR) ranging from 1.1 to 5.5. Maternal race/ethnicity was cited in eight differing studies [7, 19, 28, 30, 32, 38–40]. Compared with other race/ethnicities, newborns of Asian mothers were more likely to be readmitted following the initial hospitalization at the time of birth (OR = 1.08 to 3.17).

Nine studies reported that mothers with pre-existing or perinatal complications increased the probability of newborn readmission following discharge [3, 7, 19, 21, 27, 30, 32, 35, 39]. The most common pre-existing maternal medical conditions and/or pregnancycomplications that resulted in newborn readmissions included gestational hypertension, gestational diabetes mellitus, placenta previa, placental abruption, and prolonged rupture of membranes. In two studies, newborns whose mothers experienced delivery complications were found to be at high risk of unplanned readmission [3, 30]. One study [27] identified maternal intrapartum chemoprophylaxis for group B streptococcus was associated with newborn readmission (OR = 2.55). Another study [21] cited newborns of mothers who self-rated poor health as more like to be readmitted (OR = 5.17).

In five studies, health care utilization and family resources, including uninsured health care status, unstable family income and inadequate support for the mother following the discharge, were identified as increasing the risk of newborn readmission [3, 21, 31, 39, 40]. Other studies differed, citing mothers who received comprehensive perinatal care as more likely to experience a UHR of their newborn [33, 39].

The geographic location of both the hospital where the birth occurred and the residential address of parents was cited as risk factors by five differing studies [17, 23, 36, 40, 41]. Higher readmission rates were noted for births in non-metropolitan hospitals [22, 36]. When newborns were discharged to residential addresses associated with lower newborn mortality rates, the risk of UHR was decreased by 10% (OR = 0.9) [41]. Two other studies found that health services with protocols requiring longer length of stay for newborns following their birth [17] or health services which provided limited use of intensive phototherapy for jaundice [32] experienced higher rates of readmissions (OR = 1.22–2.31).

The maternal age was cited as a risk factor by five studies [7, 23, 32, 38, 41]. One study [32] suggested that newborns of mothers younger than 20 years were more likely to be readmitted (OR = 1.2). In comparison, three studies [7, 38, 41] identified that newborns of mothers older than 30 or 35 years were at greater risk of readmission. One study [23] also reported increased readmission for healthy term infants with older mothers (OR = 1.02).

## Newborn-related risk factors

Eleven significant risk factors pertaining to the newborns were extracted. The most frequently cited risk factors were gestation age, neonatal comorbidity, postnatal length of stay (LOS), and feeding methods.

Gestational age was the most frequently cited significant predictor of unplanned readmission for newborns with OR range from 1.18 to 9.43 [7, 17, 19, 23–25, 27, 30–32, 34–38, 42]. Generally, infants born before 37 gestational weeks were associated with an increased likelihood of readmission compared with those delivered at 39 to 40 weeks. Three studies specifically cited gestational age of 37 to 38 weeks as a risk factor for unplanned readmission as well [27, 30, 34].

Newborns who either had a medical condition at birth or developed medical conditions following their birth were associated with an increased risk of UHRs [2, 19, 20, 26–28, 30, 31, 33, 36, 40]. Medical conditions included jaundice, haemolysis, congenital abnormalities, complex/chronic conditions, sepsis, seizure, cardiac surgery, and myelomeningocele repair of newborns. Two studies cited infants who had a screening bilirubin test associated with jaundice during their hospitalization at the time of birth as significantly associated with increased risk of readmission with OR ranging from 6.66–8.76 [19, 33]. One study [27] indicated that jaundice assessed visually and documented on day two of life was a predictor of newborn readmission (OR = 2.45). Two studies [30, 31] involving newborns with neonatal abstinence syndrome (NAS), a postnatal drug withdrawal syndrome related to opioid exposure, found that newborns with NAS were more likely to be readmitted to the hospital compared to newborns without NAS (OR = 1.21–2.49).

Eleven included studies [2, 7, 22, 23, 30, 32–35, 38, 39] identified length of hospital stay (LOS) after birth as associated with increased risk of readmission; however, the results were inconsistent. Seven studies found shorter LOS (< 3 days) as associated with a higher risk of readmission for newborns delivered by vaginal or cesarean (OR = 1.2–13.8) [7, 30, 32, 35, 38, 39] or infants born late preterm and term (*P* < 0.05) [34].

Two studies [23, 33] reported that longer hospital LOS decreased the UHR rate for infants born by cesarean (OR = 0.40–0.78). Newborns who underwent cardiac surgery and stayed longer than 7 days in intensive care units were five times more likely to be readmitted [2]. One study [22], however, reported that longer LOS did not decrease 7-day readmission for late-preterm infants delivered vaginally.

Feeding methods and feeding problems were identified in nine studies [3, 17, 19, 27, 29, 32, 33, 35, 38]. Compared with bottle feeding, exclusive breastfeeding was found to contribute to an increased risk of newborn readmission in six cited studies [3, 29, 32, 33, 35, 38]. One study [19] also found newborns who experienced breastfeeding difficulties during birth hospitalization were more likely to be readmitted (OR = 1.85). One study [17] reported breastfeeding as associated with a lower rate of readmission for newborns (OR = 0.69). While one study [27] found newborns who were totally formula fed in the first 3 days of life were associated with decreased newborn readmission (OR = 0.996).

Gender was examined and reported consistently across seven differing studies. Compared to females, male newborns experienced a higher risk of unplanned readmission after birth [7, 23, 32, 36, 38–40].

Three studies [7, 32, 35] referred to newborns delivered as a vaginal or assisted vaginal birth (vacuum/forceps) as a higher risk of readmission compared to caesarean delivery. Two studies cited cesarean delivery mode as a protective factor against readmission [7, 27] (OR = 0.31–0.45).

The birth weight of newborns was also identified as a statistically significant factor in two studies. The measurement of birth weight, however, was inconsistent amongst the studies. One study [17] reported birth weight of less than 2500 g was associated with an increased risk of readmission among newborns (OR = 1.95), while another study [30] found newborns with birthweights in either the 10th or 90th percentile using national percentile ranges were more likely to be readmitted.

Two studies cited newborns’ weight at discharge as risk factors. One study [29] reported newborns with more than a 10% weight loss from birth at the time of discharge as at higher risk of readmission. This compared with one other study [33] which suggested that every 100 g of gained weight at discharge increased the risk of readmission for late-preterm newborns with hyperbilirubinemia.

## Discussion

This systematic review synthesized risk factors associated with newborn 31-day unplanned hospital readmissions following discharge from the hospital where the birth occurred. Twenty-eight studies were reviewed, and 17 significant risk factors were extracted. These included six maternal and 11 newborn-related variables. Of the 17 predictors, six were consistently cited. Four factors were maternal (primiparous, mother being Asian, vaginal delivery, maternal complications), and two factors were neonatal (male infant and neonatal comorbidities). The remaining risk factors were inconsistent across the included studies.

Newborns of mothers under 20 or over the age of 35, especially primiparous, were at greater risk of unplanned hospital readmission. This is consistent with evidence on the adverse outcomes of pregnancies conceived at extreme maternal age [43]. Adverse outcomes included preterm births or perinatal deaths as well as pregnancy complications such as gestational diabetes and pregnancy-induced hypertension [44–46].

Newborns of Asian mothers were found in this review to have up to a 3 times greater likelihood than other ethnicities of being readmitted. It is noted that almost 90% of the included studies (*n* = 25) in this review were conducted in western developed countries such as the USA, Canada, UK, Australia, and France with extensive multicultural backgrounds. Mothers of Asian ethnicity experience language and cultural barriers during hospitalization impacting their health literacy and comprehension of discharge information on caring for newborns and themselves [47]. Additionally, where there is inadequate family support for migrants there is often limited uptake of community support services following hospital discharge [48, 49].

Our review also revealed that early-term or late preterm newborns (34 to 38 weeks GA), who are physiologically immature, were often treated the same as a full-term newborn and experienced higher readmission rates [18, 44]. There is a need to implement evidence-based practice guidelines and individualized transitional care plans that include newborn and parental assessment of discharge readiness to prevent UHR.

Readmission rates associated with the LOS for newborns after their birth were inconsistent and varied from 1.3 to 6.6 days across 92 countries [50]. Since the 1970s, there has been a trend toward shortening postnatal hospital stays for mother and newborn [51]. Some studies found reduced LOS and early newborn discharge did not increase the adverse outcomes and/or readmission rate [52–54]. In contrast, others reported shorter LOS associated with newborn mortality and neonatal UHR [51, 55]. The heterogeneity of the study population may explain the lack of consensus in the different studies, such as the newborn’s GA or birth weight, mode of delivery and parity, access to maternity care, availability of follow-up services, and/or parental knowledge and competence [56]. The inconsistent results highlight the importance of discharge readiness assessment, including newborn clinical fitness for discharge and parental readiness for discharge. Therefore, the timing of discharge should be decided in conjunction with the families.

This review found that vaginal or assisted vaginal deliveries significantly increased the risk of unplanned newborn readmissions compared with cesarean section. This is opposite to evidence promoting the advantages of vaginal delivery. Compared to newborns delivered by cesarean section, those delivered vaginally were found to have an increased probability of newborn hyperbilirubinemia and jaundice [57, 58], which resulted in an increased risk of newborn readmission. The indicators for either an elective or emergency cesarean procedure are to correct maternal or fetal existing medical conditions or distress. As a result, a higher level of care is required to be provided for both mothers postoperatively and newborns. This often leads to a longer stay in the hospital than mothers who have a vaginal delivery resulting in mothers having a greater length of time to recover and establish routines with their babies [59]. Newborns delivered by cesarean were strongly associated with reduced readmissions for jaundice [7, 32, 35]. More extended hospitalization following cesarean section than vaginal birth allowed mothers and newborns to establish breastfeeding [60], which was a protective factor reducing the risk of newborn UHRs. Newborns delivered by cesarean were also strongly associated with reduced readmissions for jaundice [7, 32, 35]. Additionally, vacuum-assisted deliveries were found to be associated with neonatal bruising and/or cephalohematoma, which increased the risk of newborn readmission [61].

Six included studies cited exclusive breastfeeding as a predictor of newborn readmission, which conflicts with the evidence citing the advantages of breastfeeding. Notably, most studies citing breastfeeding as a risk factor were related to newborn readmissions with jaundice [29, 32, 33, 38]. Mothers who wish to breastfeed their newborns exclusively might encounter many challenges resulting in newborns’ low oral intake and poor weight gain. Challenges include limited professional support, advice, and access to primary care services during the initial period following discharge [62]. Insufficient oral intake of newborns can cause severe hyperbilirubinemia, which also leads to UHRs [33, 63].

This systematic review has certain limitations. Firstly, only English language papers with full-text access were considered. The majority of the included studies were conducted in the North America, Europe, and Australia; therefore, generalization of this review’s results should be made with caution considering the characteristic of the healthcare settings. In addition, a meta-analysis was not performed to synthesize the extracted risk factors due to the heterogeneity in the 28 included studies. The studies’ heterogeneity included newborns’ characters, examined variables, time period associated with UHRs, and outcomes coherence. This systematic review did not restrict newborn’s gestational age and comorbidities, which might contribute to the large variation of UHR rate of 0.2% to 39%.

## Conclusion

This systematic review confirms the diverse and complex nature of risk factors associated with newborn 31-day UHRs. Six consistently cited predictors include 4 maternal factors (primiparous, mother being Asian, vaginal delivery, maternal complications) and 2 neonatal factors (male infant and neonatal comorbidities). There is a need to promote healthcare providers’ awareness of risk factors then develop and implement comprehensive individualized hospital-to-home transition plans from the time of admission for the birth through to discharge home to reduce unplanned neonatal readmissions [64]. Transition checklists and discharge readiness assessments are recommended as strategies to reduce newborn UHRs as the quality of hospital-to-home transition of care is positively associated with the caregiver’s perception of readiness for discharge [65]. Transition assessment instruments include “Readiness for hospital discharge scale,” “Quality of discharge teaching scale,” and “Post-discharge coping difficulty scale” [66–70].

Applying identified predictive risk factors assists healthcare providers to recognize newborns at higher risk of readmission and implement preventative strategies, for example, individualized discharge planning [71]. Future research is warranted to establish standardized maternal and newborn-related variables for healthcare providers to identify newborns at higher risk of UHRs. The classification/grouping of newborn physiological conditions, such as GA, delivery mode, birth weight, and Apgar scores, should be clearly defined and standardized in future studies allowing comparisons across healthcare settings.

## Supplementary Information

Below is the link to the electronic supplementary material.

Supplementary file1 (DOCX 14 KB)

## Data Availability

Not applicable to a systematic review.

## Abbreviations

ELBW: Extremely low birth weight
GA: Gestational age
LBW: Low birth weight or low birthweight
LOS: Length of stay
NAS: Neonatal abstinence syndrome
OR: Odds ratio
UK: United Kingdom
UHR: Unplanned hospital readmissions
USA: United States of America
VLBW: Very low birthweight
