# Supplementary material for: Risk factors associated with 31-day unplanned hospital readmission in newborns: a systematic review
Source: Eur J Pediatr. 2023 Jan 27;182(4):1469–82. doi: 10.1007/s00431-023-04819-2 (PMC10167195; doi:10.1007/s00431-023-04819-2)
Supplement: Supplementary file 1 — Supplementary file1 (DOCX 14 KB) [file 431_2023_4819_MOESM1_ESM.docx]

**Appendix 1 Database search strategy**

**Ovid MEDLINE(R)**

**# Searches**

1 exp Patient Readmission/

2 rehospitali*.tw.

3 readmission*.tw.

4 (hospital adj10 readmission*).tw.

5 (unplanned adj10 readmission*).tw.

6 (patient adj5 readmi*).tw.

7 readmit*.tw.

8 re-admission*.tw.

9 (repeat* adj5 hospital*).tw.

10 1 or 2 or 3 or 4 or 5 or 6 or 7 or 8 or 9

11 exp Infant, Premature/ or exp Infant, Low Birth Weight/ or exp Infant, Newborn/

12 (newborn* or new born* or newly born or baby* or babies or premature or prematurity or preterm or pre term or preemie* or premie* or low birth weight or low birthweight or VLBW or LBW or ELBW or infant* or infancy or neonat*).tw.

13 exp Intensive Care Units, Neonatal/

14 11 or 12 or 13

15 10 and 14

**Embase**

**# Searches**

1 exp hospital readmission/

2 rehospitali*.tw.

3 readmission*.tw.

4 (hospital adj10 readmission*).tw.

5 (unplanned adj10 readmission*).tw.

6 (patient adj5 readmi*).tw.

7 readmit*.tw.

8 re-admission*.tw.

9 (repeat* adj5 hospital*).tw.

10 1 or 2 or 3 or 4 or 5 or 6 or 7 or 8 or 9

11 exp infant/

12 exp neonatal intensive care unit/

13 (newborn* or new born* or newly born or baby* or babies or premature or prematurity or preterm or pre term or preemie* or premie* or low birth weight or low birthweight or VLBW or LBW or ELBW or infant* or infancy or neonat*).tw.

14 11 or 12 or 13

15 10 and 14

**CINAHL**

**# Query**

S11 S6 AND S10

S10 S7 OR S8 OR S9

S9 newborn* or new born* or newly born or baby* or babies or premature or prematurity or preterm or pre term or preemie* or premie* or low birth weight or low birthweight or VLBW or LBW or ELBW or infant* or infancy or neonat*

S8 (MH "Intensive Care Units, Neonatal")

S7 (MH "Infant, Newborn+")

S6 S1 OR S2 OR S3 OR S4 OR S5

S5 readmit*

S4 re-admission*

S3 readmission*

S2 rehospitali*

S1 (MH "Readmission")
